# Chemical exchange saturation transfer MRI to assess cell death in breast cancer xenografts at 7T

**DOI:** 10.18632/oncotarget.25844

**Published:** 2018-07-31

**Authors:** Jonathan Klein, Wilfred W. Lam, Gregory J. Czarnota, Greg J. Stanisz

**Affiliations:** ^1^ Physical Sciences, Sunnybrook Research Institute, Toronto, Ontario, Canada; ^2^ Department of Medical Biophysics, University of Toronto, Toronto, Ontario, Canada; ^3^ Department of Radiation Oncology, University of Toronto, Toronto, Ontario, Canada; ^4^ Department of Radiation Oncology, Odette Cancer Centre, Sunnybrook Health Sciences Centre, Toronto, Ontario, Canada; ^5^ Department of Neurosurgery and Pediatric Neurosurgery, Medical University of Lublin, Lublin, Poland

**Keywords:** CEST, MRI, breast cancer, cell death, response monitoring

## Abstract

**Purpose:**

Detecting cell death and predicting tumor response early in a course of chemotherapy could help optimize treatment regimens and improve clinical outcomes. Chemical exchange saturation transfer (CEST) MRI was investigated *in vivo* to study properties that may be able to detect cancer death.

**Results:**

Using a magnetization transfer ratio (MTR) cutoff of 0.12 at 1.8 ppm was able to differentiate between viable tumor and cell death regions. Comparison of MTR values at this frequency showed significant differences (*p* < 0.0001) between viable tumor and cell death regions, matching patterns seen on histology. Using this cutoff, the mean increase in cell death index (± standard error of the mean) after chemotherapy was 4 ± 4%, 10% ± 7%, 10 ± 8%, and 4 ± 9% at 4, 8, 12, and 24 h, respectively.

**Conclusions:**

CEST MRI can detect cell death in MDA-231 xenografts but further work is needed to characterize the clinical applications of this finding. Maximum response to chemotherapy occurred at 8–12 h after chemotherapy injection in this *in vivo* tumor model.

**Materials and Methods:**

Breast cancer xenografts (MDA-MB-231) were scanned using 7 T MRI before and after chemotherapy. As a measure of CEST effect at 0.5 µT saturation amplitude, MTR values at frequency offsets of 1.8 and −3.3 ppm were evaluated. CEST signals after chemotherapy treatment were compared to cell-death histopathology of tumors.

## INTRODUCTION

Locally advanced breast cancer is an aggressive form of cancer associated with poor survival and high risk of recurrence [1]. Modern treatment approaches increasingly use chemotherapy before surgery (“neoadjuvant chemotherapy”) followed by radiotherapy [2]. The degree of tumor response to chemotherapy correlates with survival outcomes [3]. Standard response assessment uses anatomical measurements of tumor size but some cancer, unfortunately, does not respond, which may lead to 4–6 months of ineffective treatment associated with harmful side effects [4–6]. A method to detect response to chemotherapy early in a treatment course, such as by detecting cell death, would allow for a change in therapy for non-responders, potentially improving outcomes.

Various imaging techniques have been studied for their ability to predict tumor response. T_1_- and T_2_-weighted magnetic resonance imaging (MRI) can show tumor size and macroscopic tumor characteristics [7] and dynamic contrast-enhanced MRI (DCE-MRI) parameters may be predictive of ultimate treatment response for patients receiving neoadjuvant chemotherapy for breast cancer [8, 9]. Diffusion-weighted MRI (DW-MRI) can predict ultimate tumor response in multiple cancer types, including primary brain [10], and breast [11, 12] cancer, and metastatic breast cancer in the liver [13]. A significant difference in concentrations of choline-containing compounds detected using magnetic resonance spectroscopy (MRS) has been shown between breast cancer patients who respond to neoadjuvant chemotherapy and those who do not [14].

Non-MRI-based techniques have also been studied: Quantitative ultrasound (QUS) parameters correlate with cell death after chemotherapy [15–17]. Response assessment using positron emission tomography (PET) has shown reduced uptake of (^18^F)Fludeoxyglucose (FDG) tracer after chemotherapy [18]. Diffuse optical imaging techniques have also demonstrated changes in optical index, hemoglobin concentration and water percentage after chemotherapy in patients who respond to treatment [19].

Despite their promise, these techniques also have significant limitations. T_1_ and T_2_-weighted MRI can show anatomic detail but cannot reliably distinguish between tumor progression, radiation necrosis, and edema [7, 20]. Gadolinium-enhanced T_1_ MRI and DCE-MRI also require injection of contrast agents, increasing costs and requiring clinical monitoring for sensitivity reactions [7]. Concerns have been raised regarding the sensitivity and specificity of techniques like DCE-MRI [21, 22], FDG-PET [23] and DW-MRI, as early tumor changes in the latter may mimic findings of cell death [24]. MRS is limited by poor spatial resolution [25, 26].

As a result, there are no clinically used methods for detecting early tumor responses to therapy. New imaging techniques that can overcome these limitations would be beneficial. One promising modality is chemical exchange saturation transfer (CEST) MRI, which does not require injections of exogenous contrast media and could be easily integrated into existing clinical MRI protocols. Changes in CEST contrast have been linked to changes in the chemical microenvironment and relative concentration of metabolites [27] and cell death [28, 29]. CEST is also sensitive to small changes in metabolite concentration, making it a promising modality to detect changes in tumors early in a treatment course [30].

The principles of CEST contrast have been well described in the published literature [31, 32]. Briefly, the contrast from CEST originates from labile protons (nuclei of hydrogen atoms) rapidly exchanging between water molecules and solutes in an aqueous solution (correlation time t_c_∼10^10^ s) [33]. For *in vivo* systems such as tumors, the main solutes are proteins, which present a variety of chemical microenvironments for the protons, such as amide, amine, or aliphatic groups [34, 35]. The time it takes a proton to exchange from a solute molecule to a water molecule is short enough (relative to the proton’s relaxation time) that its spin state is preserved. Therefore, a large pool of protons is generated which retain the magnetic properties they possessed when bound to proteins, amplifying the magnetic properties of the proteins and the measurable CEST effect.

When a tumor is placed in an external magnetic field, a net magnetization is detected. If a pulse of radiofrequency (RF) energy is put into the system at the resonant (Larmor) frequency of a specific proton species, that energy is absorbed and the net magnetization is reduced (termed “saturation”) [30]. Plotting the net magnetization measured as a function of radiofrequency (RF) pulse frequency creates a spectrum (called a Z-spectrum) which reflects the contributions of different proton species (defined by the frequency of RF energy they absorb). Changes in relative concentrations of different solutes and chemical groups, including contributions from large semisolid macromolecules from the magnetization transfer contrast (MTC) phenomenon [36], can then be characterized and used to differentiate different tissue types [37].

We conducted a study of the CEST properties of a large sample of MDA-231 breast cancer xenografts before and after chemotherapy to investigate CEST parameters that can differentiate regions of viable tumor from cell death. We also sought to use CEST to characterize the time dependence of the xenograft response to chemotherapy.

## RESULTS

### Data analysis

Sixteen tumor xenografts were studied with 4 animals per group. All tumors were scanned before chemotherapy was administered. Animals (*n* = 2) with excessive motion were excluded from data analysis, which left three tumors at 4 h after chemotherapy, four at 8 h, four at 12 h, and three at 24 h available for analysis.

**Figure 1:**
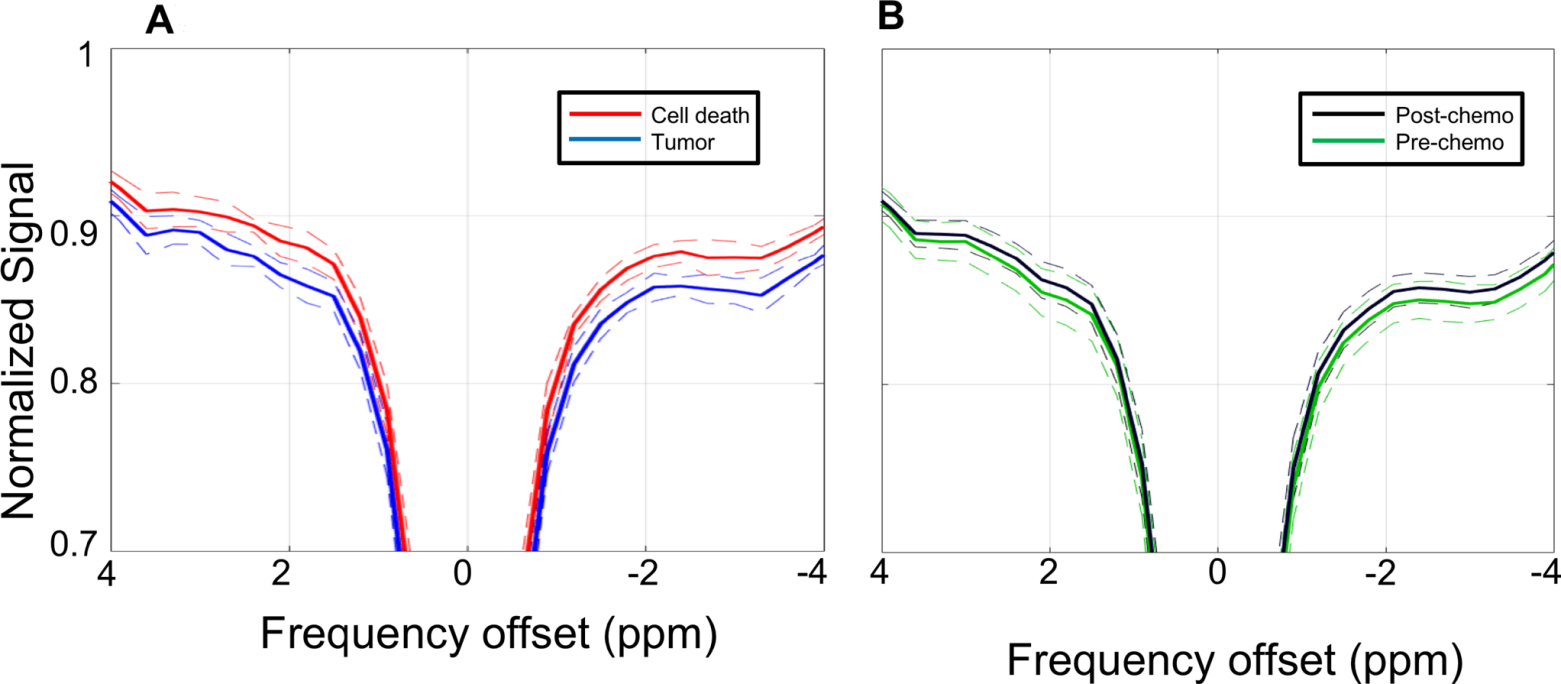
Z-spectra (**A**) Z-spectra (solid lines) averaged over the regions of viable tumor (blue) and cell death (red) as defined by visual assessment of the T_2_ structural images with co-registration of the CEST data. Dashed lines indicate standard deviations. (**B**) Z-spectra (solid lines) averaged over the entire region of interest mask for all pre-chemotherapy scans (green) and post-chemotherapy scans (black). Dashed lines indicate standard deviations.

Two initial analyses were performed. For the first, three tumors with identifiable necrotic cores were chosen based on visual assessment of the structural T_2_-weighted images. Masks were then created to estimate the areas of viable tumor and cell death. The mean Z-spectrum of the three viable tumor regions was compared to that of the three cell death regions, as shown in Figure 1A. Although large separations between the spectra were seen at 1.8, 0.6, −0.5, and −3.3 ppm, only the difference at 1.8 ppm was statistically significant in this analysis (*p* = 0.03).

The second initial analysis examined the Z-spectra of the entire xenograft region, making no attempt to differentiate between viable tumor and cell death regions. For this analysis, masks were created encompassing the entire xenograft (i.e., both areas of viable tumor and regions of cell death) based on visual analysis of the structural T_2_-weighted images. The mean Z-spectra of all pre-chemotherapy scans were then compared to the post-chemotherapy scans. As seen in Figure 1B, the difference in magnetization transfer ratio (MTR) values between these two groups were much smaller in magnitude than the differences between the areas of viable tumor and cell death compared in Figure 1A. The difference at −3.3 ppm did reach statistical significance (*p* = 0.035), while differences at other offsets such as 1.8, 0.6 and −0.5 ppm did not (*p* > 0.05).

### Defining MTR characteristics of viable tumor and cell death

**Figure 2:**
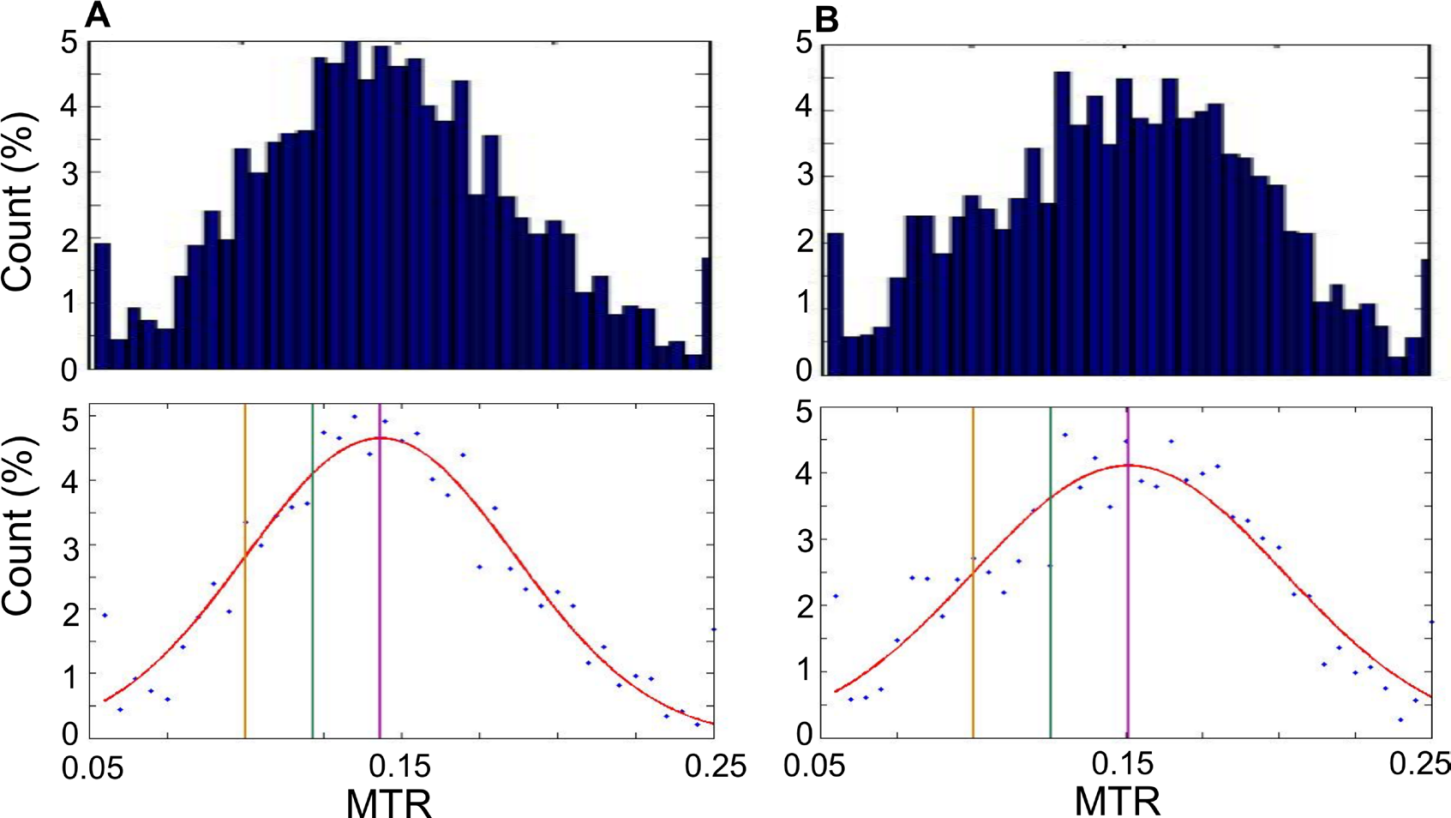
Histograms of MTR values Histograms of MTR for each pixel from all scans. MTR are counted in bins of 0.005, for 40 bins in total ranging from 0.05 to 0.25. (**A**) Histograms generated at 1.8 ppm. Top: Bar graph showing histogram data. Bottom: Scatter plot of same data as top with Gaussian curve fit to data (red). Vertical lines indicate the mean (purple; MTR = 0.14), 0.5 standard deviations below the mean (green; MTR = 0.12) and 1 standard deviation below the mean (yellow; MTR = 0.10). (**B**) As in (A) but generated at −3.3 ppm. Top: Bar graph showing histogram data. Bottom: Scatter plot of same data as top with Gaussian curve fit to data (red). Vertical lines indicate the mean (purple; MTR = 0.15), 0.5 standard deviations below the mean (green; MTR = 0.125) and 1 standard deviation below the mean (yellow; MTR = 0.10).

Based on the above results, analysis of CEST characteristics was directed toward the 1.8 and −3.3 ppm frequency offsets. Using the initial masks, which encompassed the entire area of the tumor, including any areas of cell death, the MTR was calculated for each voxel in each scan. At 1.8 ppm, the MTR for all voxels ranged from 0.076 to 0.24. At −3.3 ppm, the MTR ranged from 0.077 to 0.23. Histograms of voxel MTR values are presented in the top row of Figure 2A and 2B.

Cutoffs to label tumor and viable tissue based on the MTR were then determined. The bottom row of Figure 4 presents scatter plots of the histogram data at 1.8 ppm (Figure 2A) and −3.3 ppm (Figure 2B) offsets with several candidate tumor-cell death cutoffs defined: the mean of the distribution (labelled in purple), 1 standard deviation below the mean (1 SD; yellow) and 0.5 standard deviations below the mean (0.5 SD; green).

**Figure 3:**
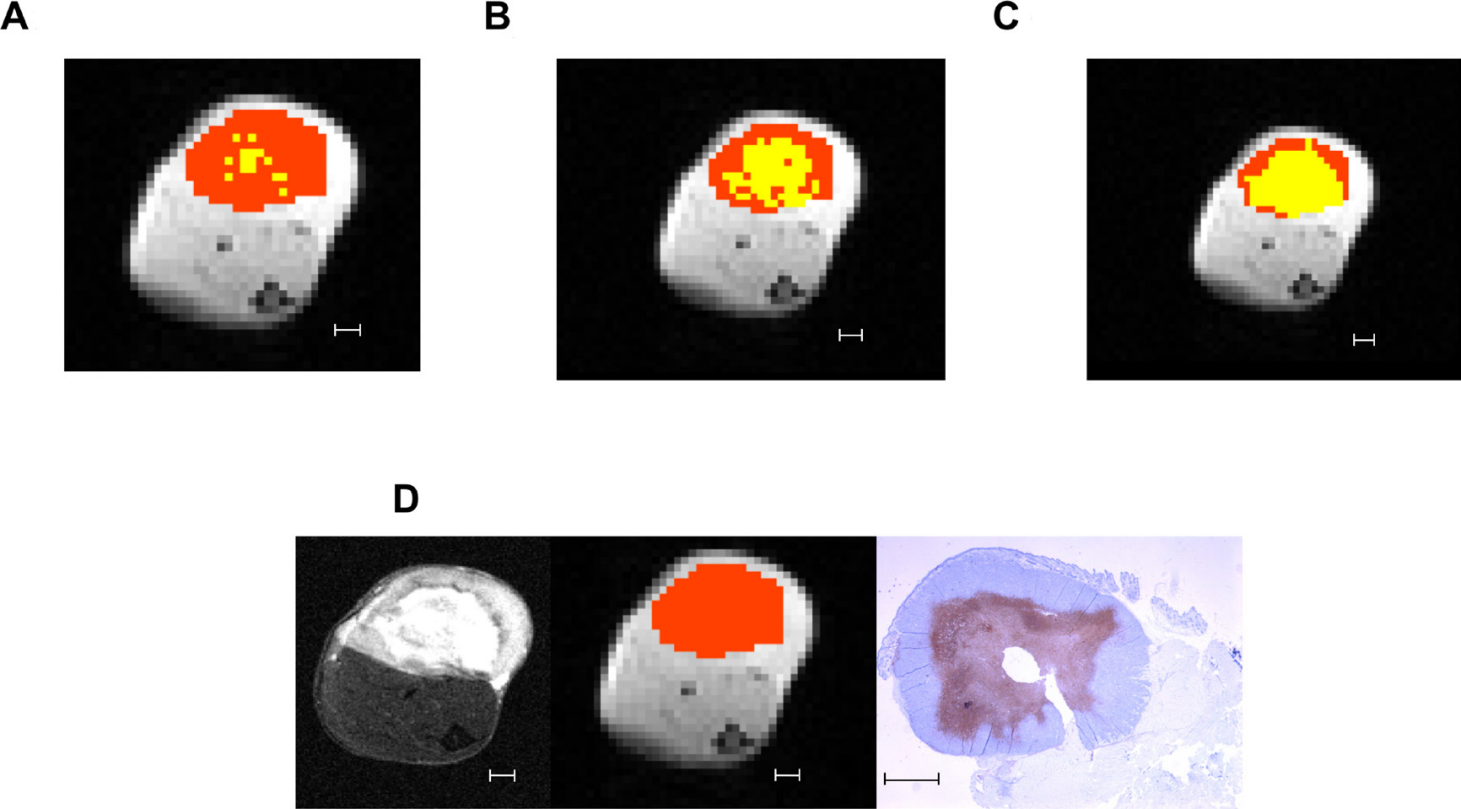
Differences in cell death regions defined at different MTR cutoffs Example of definitions of viable tumor (orange) and cell death (yellow) regions using different candidate MTR cutoffs: (**A**) 1 standard deviation below the mean (MTR = 0.10). (**B**) 0.5 standard deviations below the mean (MTR = 0.12). (**C**) Mean (MTR = 0.14). (**D**) From left to right: the T_2_-weighted structural image, pixelated CEST image with mask region overlaid in orange, and ISEL-stained histology image for reference. All scale bars indicate 1 mm.

Figure 3 presents an example of the tumor and cell death mask areas using the three different cutoffs compared with the T_2_-weighted structural image and *in situ* end labeling (ISEL)-stained histology slide for the same tumor. By visual comparison with the structural image, establishing the cutoff at the mean tended to overestimate the amount of necrosis in a tumor, while a cutoff at 1 standard deviation below the mean underestimated.

**Figure 4:**
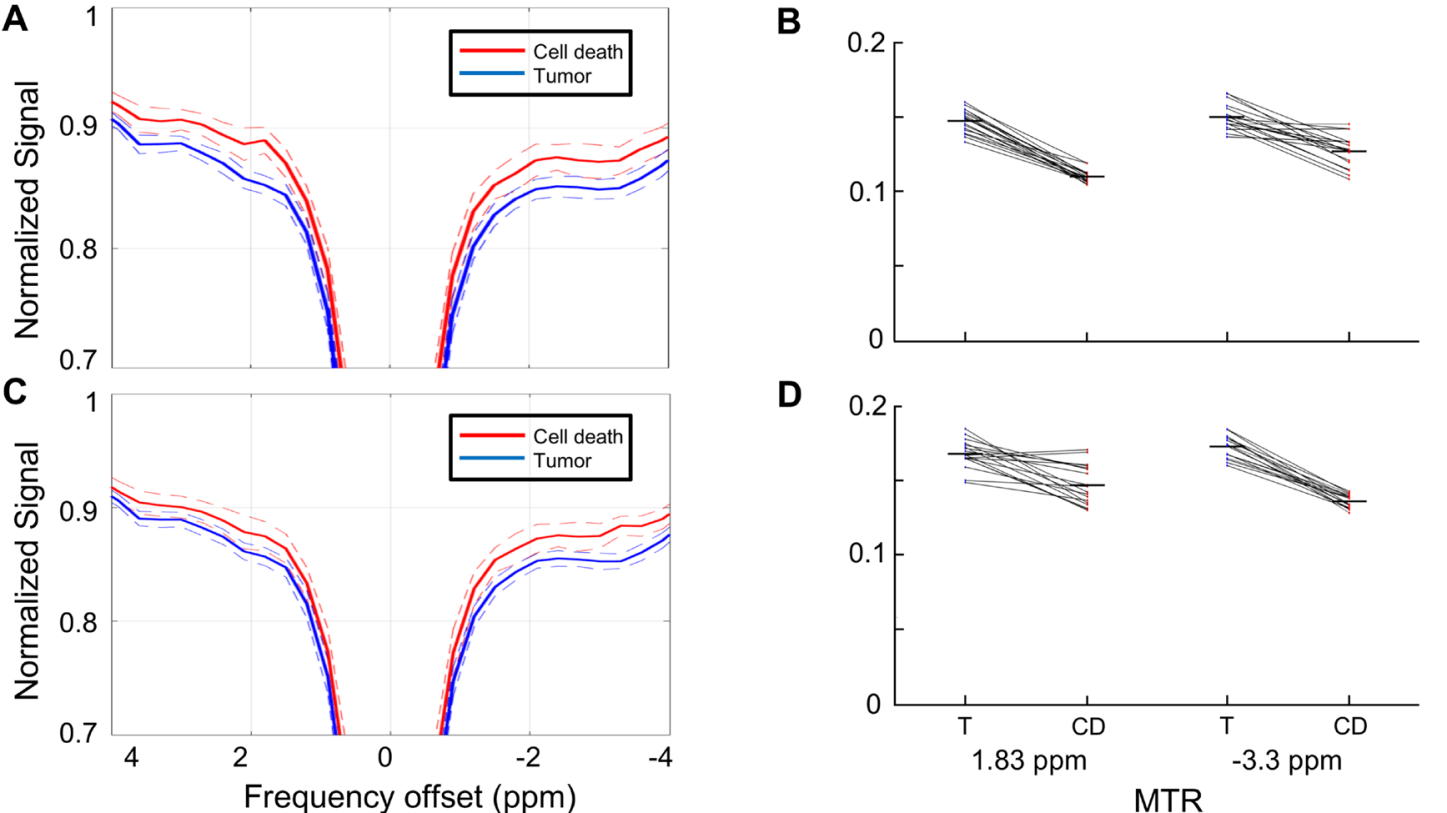
Z-spectrum comparison between cell death and viable tumor regions (**A**) Z-spectra (solid lines) averaged over the regions of viable tumor (blue) and cell death regions (red) as defined by the MTR for each voxel at 1.8 ppm using MTR = 0.12 (0.5 standard deviations below the mean of the calculated histogram) as the cutoff. Dashed lines indicate standard deviations. (**B**) Mean MTR of the masks for each individual xenograft used in Section A. The tumor and cell death masks differentiated using MTR = 0.12. The MTR difference between the masks at the 1.8 ppm and −3.3 ppm cutoffs are both statistically significant using this cutoff (*p* ≤ 0.001). (**C**) Z-spectra (solid lines) averaged over the regions of viable tumor (blue) and cell death regions (red) as defined by the MTR for each voxel at -3.3 ppm using MTR = 0.125 (0.5 standard deviations below the mean of the calculated histogram) as the cutoff. Dashed lines indicate standard deviations. (**D**) Mean MTR of the masks for each individual xenograft used in Section C. The tumor and cell death masks differentiated using MTR = 0.125. The MTR difference between the masks at the 1.8 ppm and −3.3 ppm cutoffs are both statistically significant using this cutoff (*p* ≤ 0.001). T = viable tumor regions; CD = cell death regions

Assessment of the distribution at −3.3 ppm, the offset which showed the largest separation between the pre- and post-chemotherapy mean Z-spectra (Figure 3) showed similar distributions with MTR matching those at 1.8 ppm for the different cutoffs.

### Comparison of viable tumor to cell death

The difference in CEST parameters among tumors that had identifiable cell death was examined next. Masks defining regions of viable tumor and cell death were created using the MTR map at 1.8 ppm and −3.3 ppm; the cutoff between tumor and cell death used to define these regions was set at the 0.5 SD cutoff for each offset (MTR = 0.12 at 1.8 ppm, MTR = 0.125 at −3.3 ppm).

The spectra for tumor and cell death regions are shown in Figure 4A using masks generated at 1.8 ppm and in Figure 4C using masks generated at −3.3 ppm. Regardless of which offset was used to define the masks, the maximum separation between the curves outside of the direct effect region was observed at 1.8 and −3.3 ppm. The mean MTR of the masks for each individual xenograft are shown in Figure 4B (using MTR at 1.8 ppm to define the masks) and 4D (using MTR at −3.3 ppm to define the masks). The differences in MTR were statistically significant for all cases shown (*p* ≤ 0.001).

**Figure 5:**
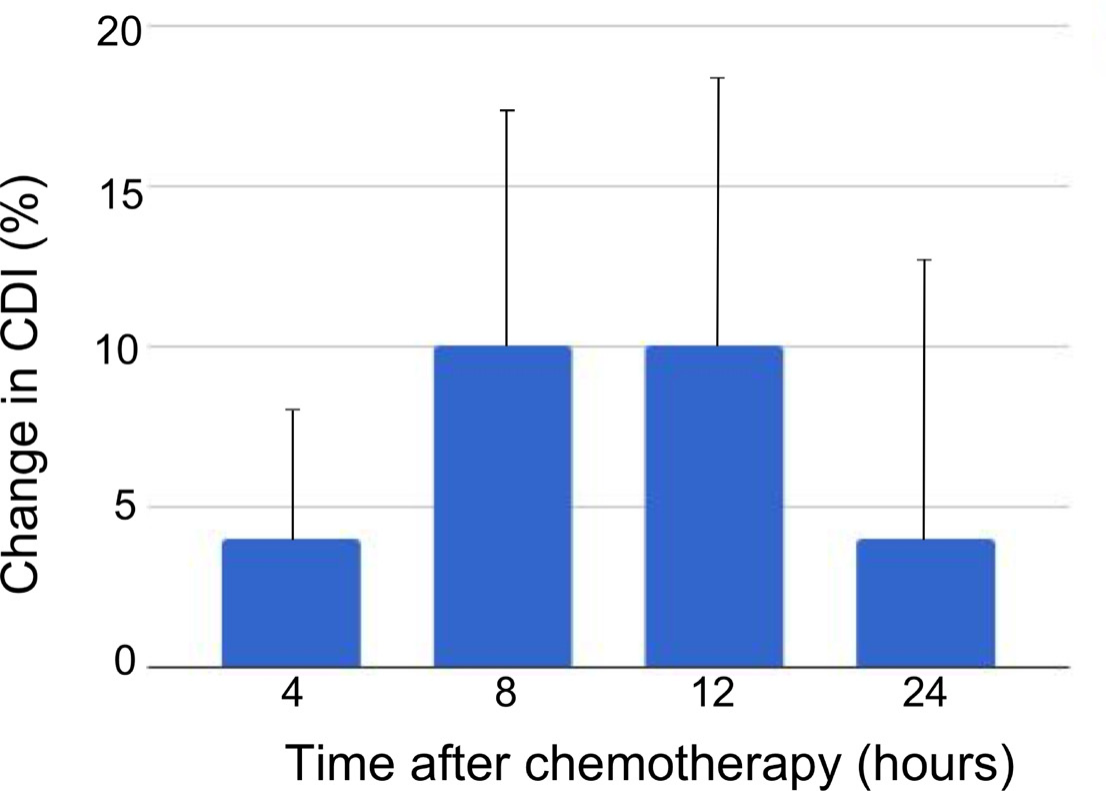
Change in cell death index by time after chemotherapy administration Average change in cell death index from pre- to post-chemotherapy scans as defined at 1.8 ppm frequency offset using the MTR = 0.12 cutoff for viable versus dead tumor. Error bars denote standard error of the mean. The differences between groups did not reach statistical significance.

Figure 5 shows the mean change in measured cell death index (CDI) as a function of time after chemotherapy. Although no differences between experimental times reached statistical significance, a trend is evident with the maximum cytotoxic effect at 8–12 h after chemotherapy administration.

## DISCUSSION

This study investigated methods for differentiating viable tumor from tumor regions containing cell death using CEST MRI. Using an MTR cutoff of 0.12 at 1.8 ppm or 0.125 at −3.3 ppm to differentiate viable tumor from cell death closely approximated the cell death pattern seen on histological assessment. Using each of these cutoffs to delineate regions containing cell death, the mean MTR of the cell death region showed a significant difference from the mean MTR of the viable tumor region. Using the 1.8 ppm cutoff to calculate CDI, a maximum increase in cell death was observed between 8–12 h after chemotherapy, after which the CDI diminished. Although this trend of increasing and then decreasing CDI over time was not statistically significant given the small number of animals at each experimental time (*n* = 3–4), it is consistent with other studies which have studied time related changes with chemotherapy using non-invasive imaging [17, 28].

Desmond *et al.* [28] used a smaller sample size of MDA tumors to observe that MTR analysis can differentiate viable tumor from cell death in this cell line. In their sample of 20 LLC lung carcinoma xenografts and four MDA breast cancer xenografts, the authors studied a variety of MRI parameters, including T_1_ and T_2_ relaxation, diffusion, and CEST parameters such as MTR and Lorentzian curve peak amplitudes corresponding to amide, amine, and aliphatic groups within Z-spectra. They compared these parameters between tumor regions which histopathological results indicated to be viable or comprised of cell death. Diffusion measurements were not significantly different between tumor and cell death regions. Differences in CEST parameters were observed between tumor and muscle. Differentiation between tumor and cell death was observed in the amplitude of Lorentzian peaks fitted to the Z-spectrum centered on the resonance frequencies of amide (3.5 ppm), amine (2 ppm) and aliphatic (−3 ppm) protons (*p* < 0.05). In our study, the largest MTR separation between tumor and cell death regions was measured at 1.8 ppm and, therefore, we directed our attention to that frequency in our study.

Previous work has also shown that amide proton transfer (APT) MRI, a CEST-based mechanism targeted at the resonance frequency of amide protons on tissue proteins (∼3.5 ppm), can distinguish radiation necrosis in the brain from normal brain tissue [38] and glioma xenografts in pre-clinical models [20]. In the latter study, APT signal changes were observed early after radiation therapy (3 and 6 days after treatment) while other imaging techniques like T_1_, T_2_, and DW-MRI showed no change at these time points [20]. Dula *et al.* applied APT MRI to a small number (3) of breast cancer patients before and after chemotherapy. They reported that APT may be useful for assessing breast cancer response to chemotherapy, as one patient who experienced progressive disease showed an increase in APT signal after one cycle of chemotherapy while the two patients who ultimately responded to chemotherapy showed APT signal decrease [7].

A clinical study of patients with brain metastases [29] used CEST to differentiate between tumor progression and radiation-induced cell death following stereotactic radiosurgery. This study showed maximum MTR difference between cell death and progressive tumor in the amide and aliphatic regions of the Z-spectra, corresponding to 3.5 and −3.5 ppm, respectively. The −3.5 ppm offset is similar to the –3.3 ppm offset with maximum separation between the pre- and post-chemotherapy Z-spectra in our study. CEST can also differentiate progression of glioma from pseudoprogression, a benign phenomenon which mimics the MRI characteristics of glioma progression after concurrent chemotherapy and radiation therapy [39].

Our study sought to investigate the entire Z-spectrum to identify regions which may provide contrast between regions of viable tumor and cell death in breast cancer xenografts. Our results suggest that the largest separation is seen around the 1.8 ppm offset, rather than the 3.5 ppm offset targeted by the APT method and identified by Mehrabian *et al.* [29]. A preliminary report of CEST MRI for breast cancer patients [40] compared CEST results at 1.2–1.8 ppm with results from DCE-MRI. In 3 of the 6 patients in this cohort, high CEST signal correlated well with tumor identified using DCE-MRI and CEST signal values were higher in tumor than in surrounding fibroglandular tissue. Our results support these findings, with significantly higher MTR values measured for viable tumor at 1.8 ppm compared with cell death regions.

Other imaging methods, aside from CEST MRI, can detect cell death *in vivo*, albeit at later stages of advanced necrosis. When these methods have been applied at varying times after treatment, a trend is evident whereby the cell-death inducing effect of the treatment increases to a point after which it begins to decrease. Tadayyon *et al.* [15] used high (20 MHz) and low frequency (7 MHz) QUS to study cell death in MDA-MB-231 xenografts using the same chemotherapy regimen used in the work here. Histological analysis showed an increase in CDI up to 24 h after chemotherapy, with the CDI at 48 h lower than at 24 h, although still statistically significantly increased over baseline. A similar pattern was demonstrated for the change in average acoustic concentration, which was highest at 24 h after chemotherapy followed by a reduction at 48 h. In a separate study [17] which treated HTB-67 melanoma xenografts with photodynamic therapy and used high frequency (26 MHz) QUS, similar patterns were observed in the parameters of midband fit and spectral slope, which have been correlated with cell death [41, 42]. The peak effect was observed between 12–20 h after treatment, followed by a decline. In the work here, the CDI calculated using the 0.5 SD cutoff to define necrotic tissue demonstrated a similar trend. The largest average increase in CDI was seen 8–12 h after chemotherapy, with the increase reduced after 24 h.

Our experimental design did introduce some limitations into this study. For example, the time required to set up and conduct each scan was approximately 2.5 hours. During this time, some tumor movement could have been experienced such as due to slow drifts in the equipment position or deflation of pads and pillows used to set up the mouse on the scanner. Image registration was employed in the fitting algorithms to minimize the effects of such motion. Registration is more accurate when multiple slices are acquired (allowing 3D registration). However, we only acquired single slices limiting registration to in-plane.

Because this experiment was primarily intended to demonstrate proof-of-concept, preparing and scanning a large number of tumors (e.g, 5 or more) per time point would take an unnecessarily large amount of time and resources, such as machine time and animal specimens. Therefore, using 3–4 specimens per post-chemotherapy time period was deemed a reasonable compromise between experimental expediency and sample size, although this did result in a relatively large variance amongst a small experimental number of animals per group.

Resource management and patient comfort considerations make long scans untenable in human trials. Reducing the number of frequency offsets used in clinical trials, for example by obtaining data from several offsets around 1.8 ppm while minimizing the data taken in other offset regions, would permit the use of shorter scans, consequently reducing scan costs and improving patient satisfaction by not requiring long periods of cooperation lying in an MRI scanner. Measurements at fewer offsets may also allow for longer RF saturation times given the availability of multiple RF amplifiers, which generally have limited duty cycles, on a clinical scanner. This data can be used to guide decisions to optimize scan protocols for future planned clinical trials.

Detection of cell death *in vivo* provides a promising avenue for early response assessment and prediction for patients undergoing neoadjuvant chemotherapy for locally advanced breast cancer. Higher MTR values are seen for viable tumor than cell death regions throughout the Z-spectrum, although the 1.8 ppm offset showed the largest separation and was the only one we studied that showed a statistically significant difference. Our ability to distinguish cell death from viable tumor using CEST imaging in this frequency region agrees with preliminary results reported by Schmitt *et al.* [40].

Our data provides proof of principle in a large pre-clinical dataset suggesting that differences in CEST effect are seen across the Z-spectrum which can detect cell death in breast cancer. However, the magnitude of the signal change is small and further work is required to define the contributions to the observed effect (e.g, increased water content, changes in guanidine concentration [43], or other factors), specificity of the observed effect to cell death, and to translate the work to clinical scenarios. Nevertheless, MTR measurements around the 1.8 ppm offset should be a point of interest in studies attempting to translate CEST MRI analysis into clinical practice and may be investigated alone or in combination with previously studied metrics such as Lorentzian peak amplitude to develop prediction algorithm based on multiple CEST parameters. Further study, in animal models or humans, can combine CEST MRI with other validated imaging modalities to further refine detection methods to detect cell death and improve predictive models for response and clinical outcomes.

## MATERIALS AND METHODS

### Animal model

Tumors were grown by injecting 100 μL of solution containing up to 5 × 10^6^ MDA-MB-231 tumor cells (American Type Culture Collection, Manassas, VA, USA) into the hind legs of CB-17 severe combined immunodeficiency (SCID) mice (Charles River Laboratories, Canada, Saint-Constant, QC, Canada).

Animal care protocols were approved by the local Animal Care Committee at Sunnybrook Research Institute. Mice were anesthetized during scanning by inducing anesthesia with 34% isoflurane. Thereafter, respiratory rate was monitored by a pneumatic pillow. Isoflurane concentration was titrated to maintain a breathing rate of 60–90 breaths per minute; generally, 1–2% isoflurane concentration was sufficient to maintain this rate. Temperature was monitored with a probe placed in constant contact with the skin of the mouse’s stomach. Constant external temperature was maintained using a warm water circulating bath.

**Figure 6:**
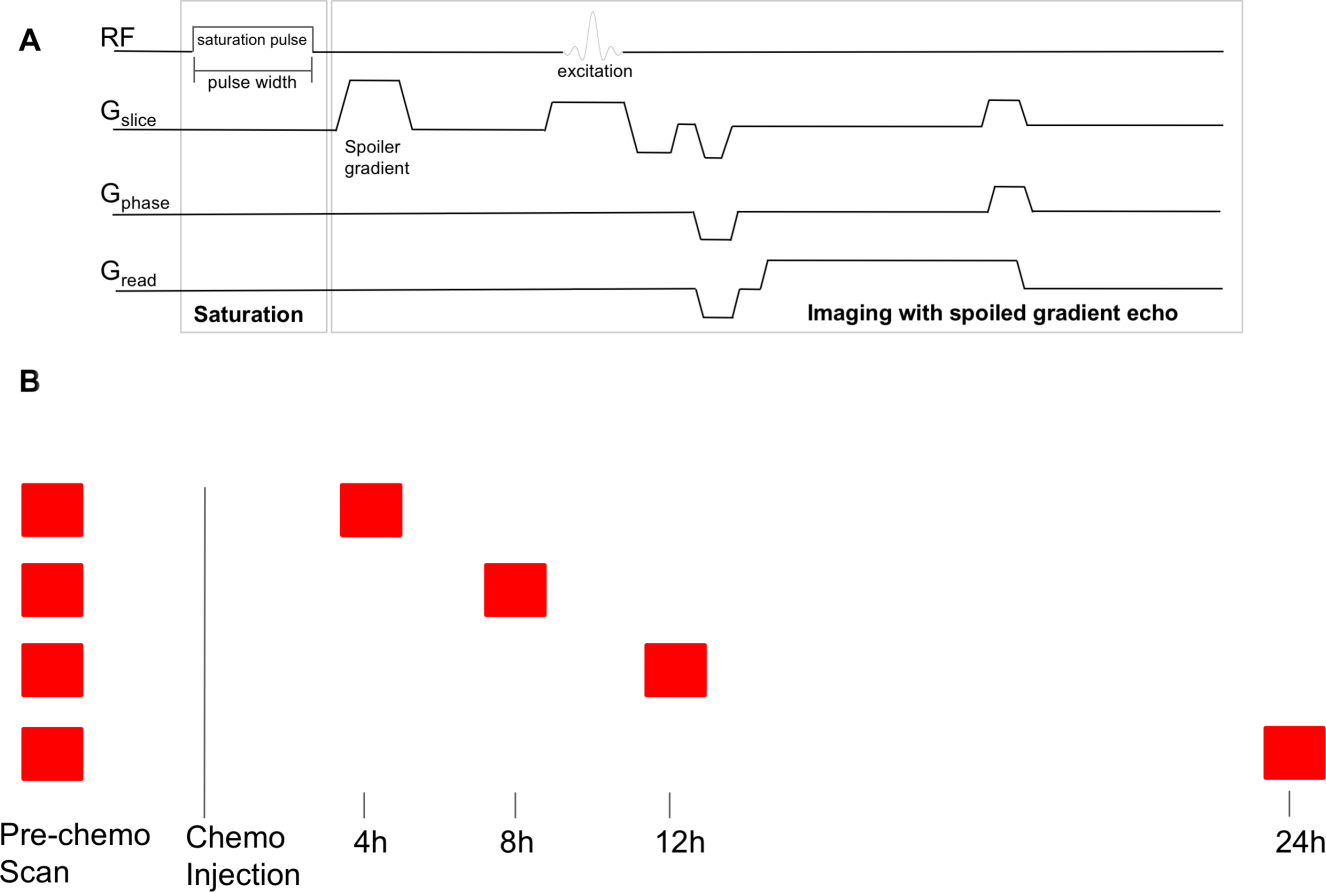
CEST MRI pulse sequence and timing of scans (**A**) CEST MRI pulse sequence. The line labelled “RF” shows radiofrequency pulse application. The lines labelled G_slice_, G_phase_, and G_read_ show the imaging gradients. Adapted from: K. L. Desmond, “Endogenous Chemical Exchange Saturation Transfer: Quantitative Modelling and Application in Cancer.” (**B**) Timing of scans. Each tumor was scanned before chemotherapy, and then either at 4, 8, 12, or 24 h after chemotherapy injection. The scan was arranged such that the appropriate time after injection occurred at some point during the CEST scan.

Due to the known propensity for MDA-MB-231 xenografts developing necrotic cores [44], tumors were scanned when they reached approximately 5 mm in diameter as evaluated by measuring the visible tumor using calipers. Doxorubicin (50 mg/m^2^) and paclitaxel (100 mg/m^2^) chemotherapy was used, as these drugs and doses form the basis of standard, modern, clinical neoadjuvant chemotherapy regimens. The chemotherapy was administered via tail vein catheter immediately after completion of each pre-chemotherapy scan. Tumors were rescanned at a pre-determined time (4, 8, 12, or 24 h) after chemotherapy injection. Previous work has shown significant changes in imaging properties of MDA-MB-231 xenografts over these time frames using this dosing strategy [45]. Scans were timed such that they overlapped with some part of the CEST scan, as illustrated in Figure 6.

Immediately after completion of the post-chemotherapy scan, animals were sacrificed under anesthesia by cervical dislocation. Tumors were excised, leaving the skin overlying the tumor and a layer of muscle underneath the tumor intact. The tumor was cut in half at the point of largest diameter. The proximal portion of the tumor tissue was fixed in 10% formalin and then transferred to a solution of 70–80% ethanol for storage until processing; the distal portion was frozen in liquid nitrogen and stored in an 80° C freezer for future retrieval. The proximal portion of the tumor was sectioned into 5 μm slices and stained with hematoxylin and eosin (H & E) for morphological identification and ISEL assay for identification of cell death (apoptosis and necrosis). As apoptosis and necrosis are both detectable using ISEL staining, this report refers to regions stained by ISEL as regions of “cell death” [46, 47].

### MRI imaging

Animals were imaged before and after chemotherapy injection on a 7 T preclinical MRI system (BioSpec 70/30 USR, Bruker BioSpin, Billerica, MA). A volume coil was used for transmission and a 20-mm diameter surface coil was used for reception. The tumors were positioned at the isocenter of the magnet for optimal shimming. A high-resolution, T_2_-weighted Rapid Acquisition with Relaxation Enhancement (RARE) image [48] (RARE factor 8, TR/TE = 2500/50 ms) was acquired with 11 slices and the tumor volume identified to perform field map-based shimming using Bruker’s Map Shim functionality. A correction to account for spatial inhomogeneity in the B_0_ field was also performed [49].

The MRI sequence used began with a single rectangular off-resonance RF pulse of 490 ms which was followed by a single slice 2D FLASH sequence with TR/TE = 501/3.1 ms at a resolution of 0.31 mm × 0.31 mm × 1 mm and a matrix size of 64 × 64. Saturation pulse amplitude of 0.5 µT was used. The MRI pulse sequence is shown in Figure 6. Measurements were made at frequency offsets between −1800 Hz (−6 ppm) and 1800 Hz (6 ppm) in increments of 30 Hz between −180 Hz (−0.6 ppm) and 180 Hz (0.6 ppm) and increments of 90 Hz outside this region. Reference images at 200 kHz offset were interleaved every 5 offsets throughout the acquisition to correct for signal drift. With the described protocol, scanning each xenograft took approximately 2 hours. While previous signal drift reports showed exponential decay of the reference signal over time, [49] our decay showed linear characteristics, which were used for the correction methods.

### Region of interest definition

To define the regions of interest for analysis, the structural and CEST images were co-registered. An area encompassing the tumor, as visualized on the structural image, was manually delineated on the CEST image. An example is shown in Figure 8, including the corresponding H & E and ISEL stained histology slides. This area (the “mask”) was intentionally drawn conservatively to ensure that the mask remained within the tumor over the entirety of the scan, accounting for small amounts of motion over the length of the scan.

The MTR was then calculated for each voxel within the masks for a given frequency offset. The voxels were then assigned as cell death or tumor based on the MTR. Once the mask was defined, a histogram was created by assigning each voxel into bins by MTR at a given frequency offset. The histogram was fit to a Gaussian distribution to define cutoffs to segment the masks in viable tumor and necrotic/apoptotic tissue. Using these masks, the CDI was calculated by the formula

$$CDI=\frac{N_{below}}{N_{total}}$$

Where N_below_ is the number of voxels with MTR below the cutoff (indicating the presence of cell death) and N_total_ is the total number of voxels within the mask encompassing the tumor. The statistical significance between differences in MTR was tested using paired *t*-tests to compare pre- to post-chemotherapy scans and using unpaired *t*-tests to compare viable tumor to cell death.

### Z-spectrum metrics

**Figure 7:**
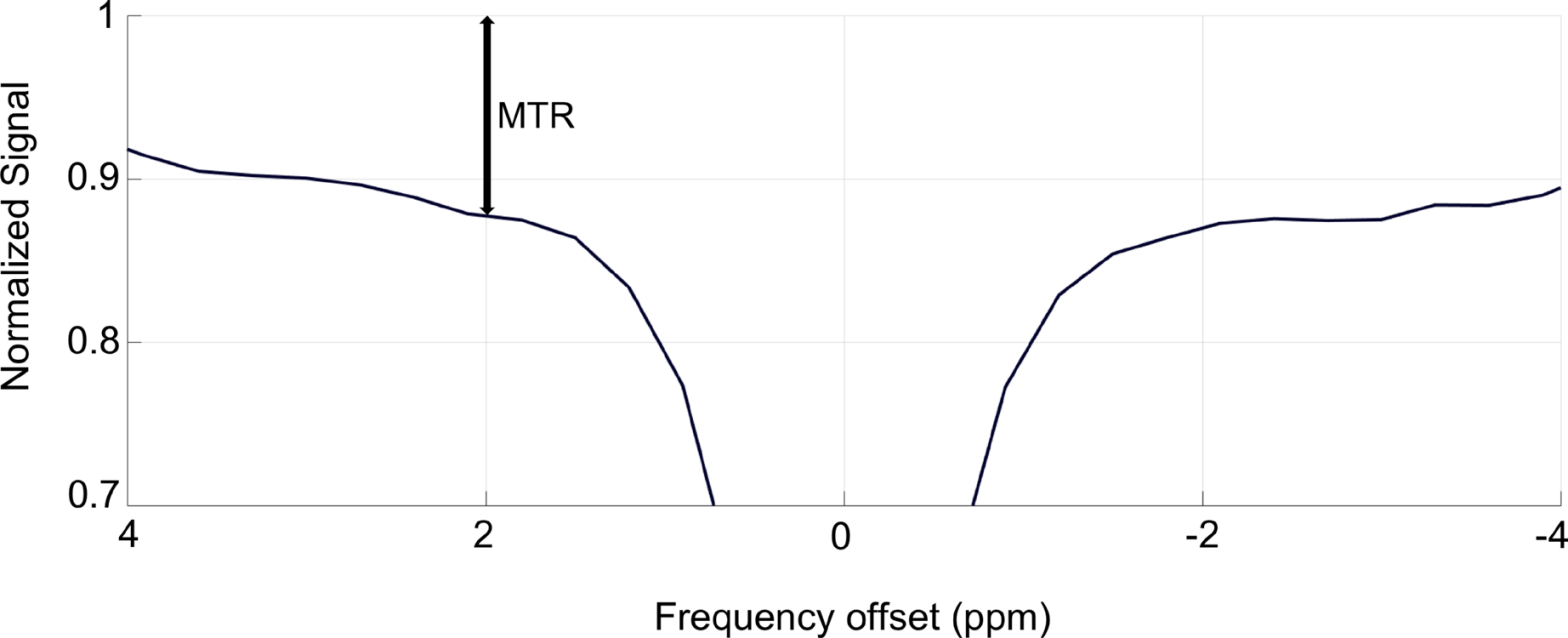
Graphical representation of the value of the magnetization transfer ratio (MTR) This figure shows the MTR value at the 2 ppm offset of a sample Z-spectrum.

Z-spectra were compared using the magnetization transfer ratio (MTR) metric, which combines the contributions from all contrast mechanisms (including CEST and MTC). The MTR is defined as

$$MTR=1-\frac{S}{S_{0}}$$

where S is the measured strength of the MRI signal at a specific frequency offset of RF saturation and S_0_ is the strength of the MRI signal when no RF saturation is applied [36]. The ratio represents $\frac{S}{S_{0}}$ the “normalized signal.” A graphical representation of the value of the MTR is shown in Figure 7. Signal strength measurements like “MTR asymmetry” are less appropriate to use in this experiment due to the inherent assumption that non-CEST contributions to the Z-spectrum are symmetric around the water signal which is not true for a complex, *in vivo* system such as a breast cancer tumor [28, 30].

**Figure 8:**
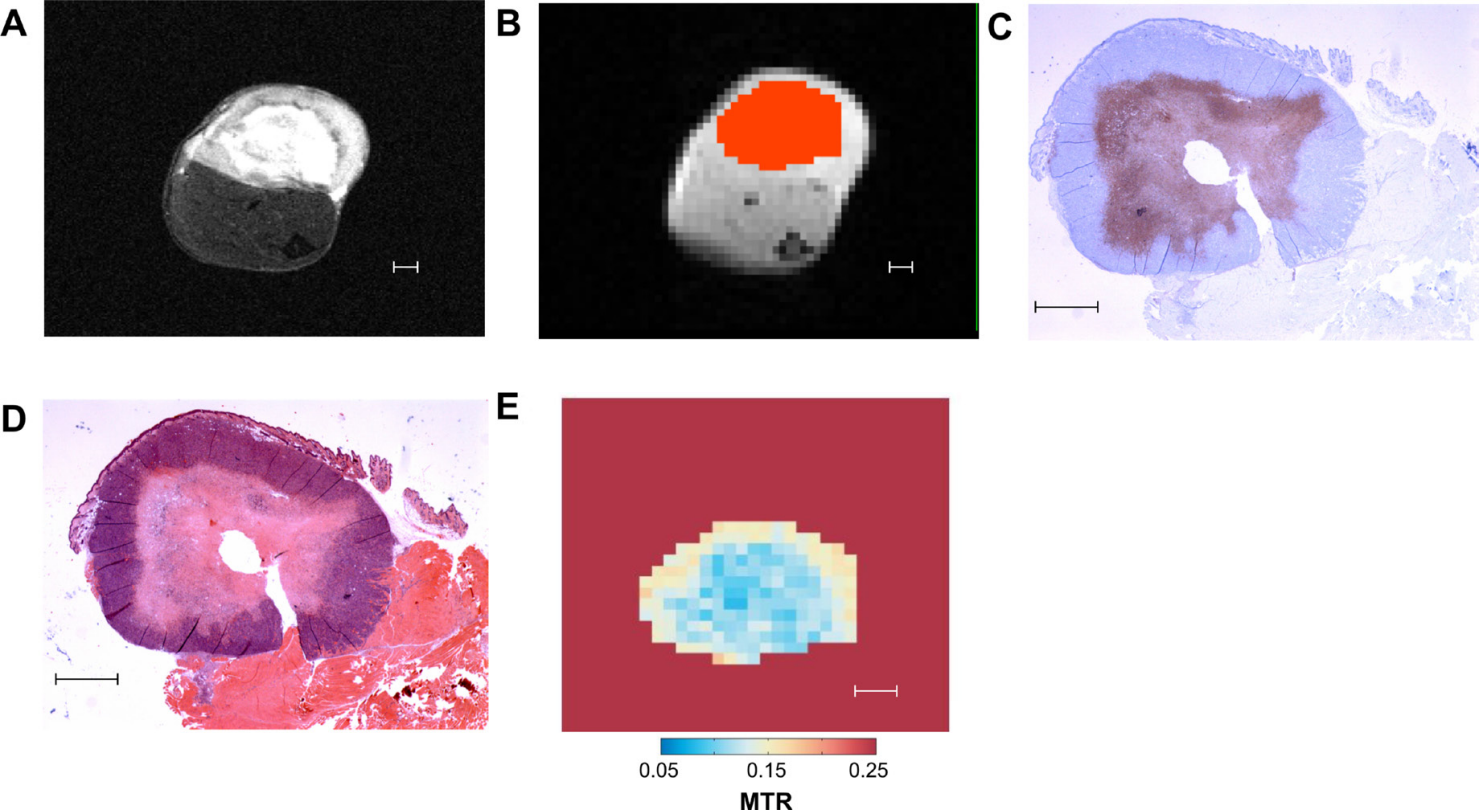
Representative images of different methods of tumor analysis employed in our study (**A**) T_2_-weighted “structural” MRI image. (**B**) CEST MRI image divided into pixels for analysis. Overlaid in orange is the mask defining the region of interest for CEST analysis. (**C**) ISEL stained histology slide: blue indicates viable tumor, purple indicates cell death. (**D**) H & E stained histology slide. (**E**) Map of MTR for each mask pixel at 1.8 ppm frequency offset. All scale bars indicate 1 mm.

The frequency, in hertz, at which protons resonate is a function of the external magnetic field strength (e.g, in an MRI machine), given by the formula

$$f_{0}=\frac{\gamma }{2\pi }B_{0}$$

where f_0_ is the resonant frequency γ, is the gyromagnetic ratio (a constant specific to a given proton species), and B_0_ is the magnetic field strength (7 T in this experiment). Larmor frequencies are generally (as in this study) reported in units of parts per million (ppm), which is independent of MRI field strength, and given by the formula

$$\Delta =\frac{f_{0}-f_{0,\text{ref}}}{f_{0,\text{ref}}}\times 10^{6}$$

where Δ is the frequency offset (in ppm), f_0_ is the resonant frequency of interest, and f_0,ref_ is the resonant frequency of a reference compound. Using units of ppm allow for accurate comparison of data collected using experimental setups with different B_0_ values. The convention in CEST analysis is to use water as the reference (contrary to MRS, which uses tetramethylsilane as the reference), so the frequency offset of water is defined as 0 ppm. In this report, Z-spectra were plotted with frequency represented in ppm.

## Abbreviations

APT: amide proton transfer
CDI: cell death index
CEST: chemical exchange saturation transfer
DCE-MRI: dynamic contrast enhanced magnetic resonance imaging
DW-MRI: diffusion-weighted magnetic resonance imaging
FDG: (^18^F)Fludeoxyglucose
H & E: hematoxylin and eosin
ISEL: *in situ* end labeling
MRI: magnetic resonance imaging
MRS: magnetic resonance spectroscopy
MTC: magnetization transfer contrast due to semisolid macromolecules
MTR: magnetization transfer ratio
PET: positron emission tomography
QUS: quantitative ultrasound
RARE: Rapid Acquisition with Relaxation Enhancement
RF: Radiofrequency
SCID: severe combined immunodeficiency
